# Amputations of Lower Limb in Subjects with Diabetes Mellitus: Reasons and 30-Day Mortality

**DOI:** 10.1155/2021/8866126

**Published:** 2021-07-24

**Authors:** Magdalena Walicka, Marta Raczyńska, Karolina Marcinkowska, Iga Lisicka, Arthur Czaicki, Waldemar Wierzba, Edward Franek

**Affiliations:** ^1^Department of Internal Diseases, Endocrinology and Diabetology, Central Clinical Hospital of the Ministry of the Interior and Administration, Warsaw, Poland; ^2^Department of Human Epigenetics, Mossakowski Medical Research Centre, Polish Academy of Sciences, Warsaw, Poland; ^3^Department of Analyses and Strategies, Ministry of Health, Warsaw, Poland; ^4^Satellite Campus in Warsaw, University of Humanities and Economics in Lodz, Poland

## Abstract

**Background:**

Diabetic foot is one of the leading causes of patient disability worldwide. Lower-extremity amputations (LEAs) resulting from this disease massively decrease quality of life, the function of the patient, and incur significant healthcare costs. The aim of this study was to assess trends in the number of amputations, the diagnosis at discharge, and diagnosis-related mortality after LEA procedures in a nationwide population.

**Methods:**

Datasets of the National Heath Fund containing information about all services within the public healthcare system in Poland, spanning the years 2010-2019, were analyzed. The source of data regarding mortality was the database of the Polish Ministry of Digital Affairs.

**Results:**

Between 2010 and 2019, the annual number of amputations in patients with diabetes increased significantly from 5,049 to 7,759 (*p* for trend < 0.000001). However, the number of amputations in patients with diabetes calculated as a number per 100,000 diabetics decreased significantly (*p* for trend < 0.0005) during this period. Amputations in patients with diabetes accounted for a majority of all amputations; the mean percentage of amputations in patients with diabetes was 68.6% of all amputations (from 61.1% in 2010 to 71.4% in 2019, *p* for trend < 0.0000001). The most common disease diagnosed at discharge after LEA in diabetic patients was diabetes itself. Vascular pathologies, such as soft-tissue/bone/joint infections and ulcerations, were the next most common. The 30-day mortality rate after LEA was rather high in patients with, as well as without, diabetes (depending on the cause for amputation 3.5-34% and 2.2-28.99%, respectively).

**Conclusions:**

The number of LEA in patients with diabetes in Poland increased substantially between 2010 and 2019 along with an increasing number of diabetics. Vascular pathologies, infections, and ulcerations were the most common causes of LEA. The 30-day mortality rate after amputation was rather high and varied depending on the diagnoses at discharge.

## 1. Introduction

Diabetes mellitus is one of the fastest growing public health concerns. The prevalence of diabetes has increased in recent decades, in most developed and developing countries. Data from the Global Burden of Disease Study 2017, assessing the global, regional, and national burden and trend of diabetes in 195 countries and territories, indicate that the 2017 global prevalence of diabetes was 476.0 million. This number is expected to increase to 570.9 million in 2025 [1]. The number of patients with diabetes is also increasing in Poland—in 2014, there were 2.113 million cases, followed by 2.533 million cases in 2017 [2].

Chronic hyperglycaemia, associated with poorly controlled diabetes, causes damage to various organs and systems and induces chronic diabetes complications, leading to incapacity, reduced quality of life, and ultimately death. One of the most common complications of diabetes is a diabetic foot. Pathologies that are risk factors for the occurrence of a diabetic foot occur quite common in diabetic patients, e.g., in a study conducted in Wroclaw (Poland), it was found that 7.28% of diabetic patients have peripheral neuropathy; 35.37%, calluses; 24.2%, foot deformities; and 17.39%, features of the pathology of arterial vessels [3].

Diabetic foot syndrome may result in lower-extremity amputation. According to the Global Burden of Diseases, Injuries, and Risk Factors Study (GBD), in 2016, about 131 million people (1.8% of the global population) had diabetes-related lower-extremity complications, including 6.8 million amputations [4]. It should be noted that cases with diabetes account for 60-70% of all lower-extremity amputations (LEA) [5, 6]. Diabetes-related lower-extremity complications are a large and growing contributor to the disability burden worldwide [4]. Lower limb amputation concurrently leads to an increase in illness-related costs and a huge change in the quality of life and function of the patient. After LEA, patients have a diminished quality of life compared to the general population [7]. A review of studies from India indicates that the prevalence of psychiatric disorders among this group of patients can be in the range of 32% to 84%, including depression rates of 10.4%–63% and posttraumatic stress disorder of 3.3%–56.3% [8]. Another problem to consider in this population is the incidence of phantom limb pain and residual stump pain.

Lower-extremity amputations are also related to significant early and long-term postoperative mortality. In a national study performed in New Zealand on individuals diagnosed with diabetes, more than 11% of patients who underwent major amputation died within 30 days, whereas nearly 18% died within 90 days [9]. In another population-based cohort study conducted in Italy, including patients with diabetes undergoing a primary amputation, mortality rates at 1 and 4 years were 33% and 65%, respectively, for major LEA and 18% and 45% for minor LEA [10].

The aim of this study was to assess trends in the number of amputations, the reasons for them, and the diagnosis-related mortality after LEA procedures in patients with diabetes compared to the nondiabetic population.

## 2. Methods

### 2.1. Data Sources

The source of health-related data is the Polish National Health Fund. The analyzed datasets contain information about all services within the public healthcare system in Poland, spanning the years 2010-2019. The data contains an anonymized patient identifier, which allows the analyses to be made at the individual level. Each service within the public healthcare system (for example, hospitalizations, which were assessed in this paper) has an assigned ICD-10 code, which is put into the system by the healthcare professional who treated the patient. Additionally, in the case of surgical hospitalizations, information about surgical procedures performed within the service (like hospitalization) is also introduced into the system. The source of data regarding mortality is from the database of the Polish Ministry of Digital Affairs. The information is based on death certificates given out by registry offices; however, only the date of death, but not the cause, is accessible in this database. Both databases contain the same patient identifier, and therefore, the information can be merged. The source of Poland's population data is Statistics Poland.

### 2.2. Definition of the Diabetic Population

A person was considered diabetic when he/she had at least one diabetic ICD-10 code (any code within the E10-E14 range) reported as the main reason for the service within the public healthcare system. The registered diagnosis date was identified as the date of the very first issuance of a relevant diabetic ICD code. The diabetic population in a given year was defined as the registered prevalence at the end of the year (December 31st). Specifically, the population was composed of diabetic patients who had been diagnosed up until the end of that year and had not yet died.

### 2.3. Amputations and Diagnoses at Discharge

The procedures analyzed in this paper are amputations of the lower limb (either of feet, toes, parts of feet, or other below-knee amputations). All of the ICD-9 procedures used in the database search are listed in Table 1. The diagnoses at discharge after the LEA procedure were categorized by ICD-10 codes, which during the examined years (2010-2019) were reported as the main diagnosis for the hospitalization during which the amputation procedure was performed. The specified categories were diabetes, vascular (atherosclerosis and gangrene), sepsis, acute conditions, infections of soft tissues (skin, subcutaneous tissue, or muscles), ulcerations, infections of bones or joints, trauma (including burns and frostbites), neoplasms, or others. All ICD codes that were included into a specific category are given in Table 1.

Diabetic amputations were defined as amputations which were performed on diabetic patients, no earlier than 30 days before the diabetes diagnosis. In other words, amputations predating the diabetic diagnosis by up to 30 days were considered diabetic. This is because of the relatively common situation where the diabetic foot diagnosis is the first manifestation of a patient's diabetes. On the contrary, nondiabetic amputations were defined as amputations in patients never diagnosed with diabetes. In effect, these parameters excluded patients, in whom diabetes was diagnosed more than 30 days after the amputation, from the analysis. The number of patients excluded by these parameters totalled 2,710 during the 10 years of being analyzed.

### 2.4. Statistical Analysis

The absolute number of all amputations and the number of amputations per 100 thousand inhabitants was determined. In the population with diabetes, the number of amputations per 100 thousand diabetic patients was also determined. The percentage of diabetes and nondiabetes amputations was calculated. The statistical significance for trends was assessed using an extended Mantel-Haenszel chi-square test for linear trend [11].

## 3. Results

### 3.1. Number of Amputations

**Figure 1:**
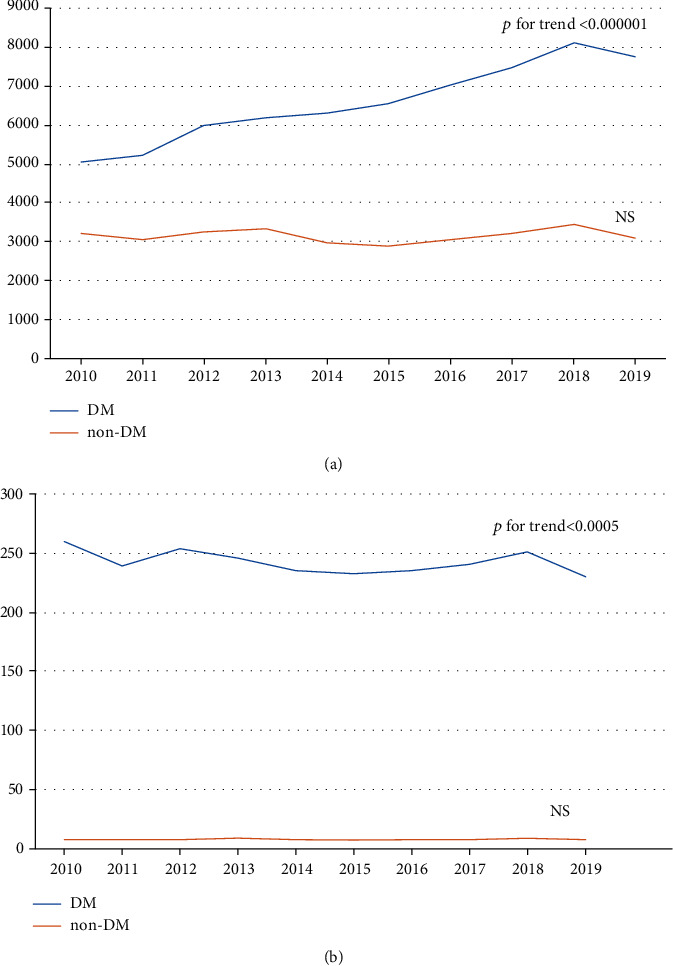
(a) Absolute number of amputations in patients with and without diabetes, and (b) number of amputations in patients with diabetes per 100,000 diabetics, and in patients without diabetes per 100,000 inhabitants in the years 2010-2019.

The number of amputations in patients with diabetes increased substantially between the years 2010 and 2019 (from 5,049 to 7,759, *p* for trend < 0.000001). In comparison, the number of amputations in patients without diabetes was stable (3,214 in 2010 and 3,109 in 2010, not significant). The trends for the number of amputations in diabetic and nondiabetic populations are shown in Figure 1(a). However, the number of amputations in patients with diabetes calculated as a number per 100,000 diabetics decreased significantly during the 10-year period (259.73 in 2010 and 229.99 in 2019, *p* for trend < 0.0005) while the number of amputations in patients without diabetes calculated per 100,000 inhabitants was stable (8.34 in 2010 and 8.1 in 2019, not significant). Those trends are illustrated in Figure 1(b).

The mean percentage of amputations in patients with diabetes accounts for 68.6% of all amputations. This number slowly increased year over year, beginning at 61.1% in the year 2010 and reaching 71.4% in the year 2019 (*p* for trend < 0.0000001).

### 3.2. Diagnoses at Discharge

**Figure 2:**
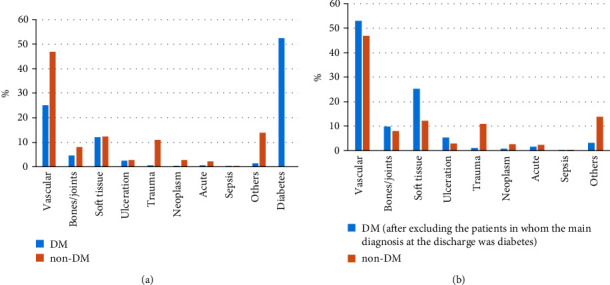
(a) Percentage number of ICD-10 categories given as the main diagnosis at the discharge from the hospital after the amputation in all study population and (b) in population after excluding the patients in whom the main diagnosis at the discharge was diabetes.

In patients with diabetes, the most common diagnosis upon discharge from the hospital was “diabetes” (see Table 1 and Figure 2(a)). This diagnosis does not give a precise reason for an amputation, and therefore, the proportion of the remaining diagnoses is altered. A decision was made to show the percentage of the diagnoses used in patients with diabetes which excluded the patients mentioned above (Figure 2(b)).

Furthermore, the proportion of patients in whom an amputation was performed for vascular reasons was similar in diabetics and nondiabetics. The same was true for both bone and joint infections. However, amputations in patients with diabetes were performed more frequently because of soft-tissue infections and ulcerations. On the other hand, trauma and other causes for amputation were more common in patients without diabetes. Sepsis was a rare cause for amputation in both groups, while neoplasms and various acute conditions (see Table 1) were in our opinion, rather concomitant diseases or complications of the procedure, than a reason for amputation (Figures 2(a) and 2(b)).

### 3.3. Thirty-Day Mortality

The 30-day mortality was rather high. The range varied depending on the reason for amputation, from 3.46 to 34% in patients with diabetes, to 2.24 to 28.99% in patients without diabetes (Table 2). In both groups, mortality was highest in patients with sepsis or acute conditions. In other cases, the 30-day mortality did not exceed 10%.

## 4. Discussion

In this paper, we have analyzed trends in lower limb amputations in patients with and without diabetes between the years 2010 and 2019 using a large national database. We found that the crude number of amputations in patients with diabetes increased substantially (over 50%) and significantly, whereas the number of amputations in patients without diabetes was stable. We have noted also that if the number of amputations in patients with diabetes was shown as a number per 100,000 diabetics, the amputation rate did not increase but rather decreased significantly during the 10 years of being analyzed. Thus, the reason for the increase in the absolute number of diabetic amputations was the increasing number of patients with diabetes.

The rise in the total number of diabetic amputations was observed also in Spain in 2002-2012 [12], but this large national Spanish study does not present the data in relation to the number of diabetic patients (in this country, the diabetes incidence also rises [12, 13]). In the nationwide study performed in Belgium between 2009 and 2013, just like in our study, the number of LEA significantly declined in individuals with diabetes and remained stable in the population without diabetes [14]. In turn, in the Irish study, performed in 2005-2009, both total diabetes-related and total nondiabetes-related amputation rates did not change significantly [15]. Similarly, in Austria, major lower-extremity amputation rates in diabetic patients remained stable between 2014 and 2017 [16].

It should be emphasized, that generally, the incidence of lower extremity amputation for all reasons in European countries is variable [17]. Even in Poland, the geographic variability of the numbers of major nontraumatic lower limb amputations in diabetics was observed [18]. The number of amputations in every country depends on many factors, i.e., total funding for healthcare, availability of specialists' clinics and highly specialist treatment [18], dedicated wound services and foot care services delivery [19], educational level, and income of patient [20].

In our study, “diabetes” was the most common diagnosis used upon discharge of diabetic patients undergoing LEA from the hospital. As mentioned above, it is obvious that this diagnosis does not identify the precise reason for amputation but rather is a diagnosis at discharge, and therefore, the proportion of the remaining reasons for diagnoses is altered. Therefore, we have decided to show, additionally and separately, the percentage of the diagnoses used in patients with diabetes excluding the patients mentioned above (Figure 2).

Unfortunately, as in more than 50% of patients with diabetes, the diagnosis at discharge was only “diabetes”; we were not able to determine the real reasons for amputation in those subjects. We also cannot be sure that a diagnosis at discharge was a reason for amputation. Those issues may be regarded as limitations of this study.

It seems however that the main cause of LEA in diabetics is vascular pathology, mainly defined as a discharge diagnosis of atherosclerosis. The proportion of patients in whom amputation was performed for vascular reasons was similar in diabetics and nondiabetics, when the discharge diagnosis of diabetes was excluded. The same was true for bone or joint infections. However, it seems that amputations in patients with diabetes were performed more frequently due to soft-tissue infections and ulcerations. This is of course not surprising, as a history of foot ulcers, osteomyelitis, or gangrene is a well-known risk factor for amputation in diabetes [21]. In the study performed in South Africa, infection and ulcers were the leading causes of LEA in diabetic patients, while ischemia was the most dominant cause in nondiabetic patients [22]. In India, infection was also found to be the leading cause of amputation [23]. However, it should be emphasized that peripheral arterial disease is reported in up to 95% of people with diabetes receiving lower limb amputations [24]. It was shown that amputation risk increases with increasing comorbidity burden, with peripheral vascular disease being one of the major independent risk factors [25]. This seems to be consistent with our study, as atherosclerosis and gangrene are more frequent causes of amputations in diabetes patients, whether the discharge diagnosis “diabetes” was excluded or not.

The 30-day mortality in our study was rather high. The range varied depending on the reason for amputation, from 3.46 to 34% in patients with diabetes, to 2.24 to 28.99% in patients without diabetes. In both groups, mortality was highest in patients with sepsis or acute conditions. We do not have the data regarding the time sequence of the amputation procedure and the diagnosis of sepsis or an acute condition. However, it seems as though those may be a consequence of, rather than a reason for, the procedure, seeing as generally such conditions are at least relative contraindications for surgery. Although there were some differences in mortality rates between diabetic and nondiabetic patients, we do not regard them as clinically significant, as in some diagnoses at discharge categories, mortality is bigger in patients with and in other ones in patients without diabetes and the percentage differences are rather modest. There does not seem to be any regularity with regard to these results.

Multiple comorbidities in diabetic patients are reasons for the increased risk of adverse events, including mortality, in this population. Therefore, it is not surprising that patients with diabetes who underwent surgery have higher risks of complications and mortality compared with patients without diabetes [26–28]. The overall 30-day mortality after major LEA reported in other studies from various countries ranged from 1% to 13.5% [9, 16, 29] and was significantly correlated with age and age-adjusted comorbidity [16]. It has also been shown that after LEA, patients with diabetes had an increased risk of death compared to nondiabetic patients [30]. However, in Ireland, a study performed in a single tertiary referral centre for vascular surgery showed no statistically significant association between mortality rate and comorbid diabetic mellitus in patients who underwent major lower limb amputation [31].

Our study has several limitations. The first one is its retrospective character. Because of this, it is difficult to assess the real reason for each amputation, especially in the patients diagnosed with “diabetes” at discharge, as well as in those for whom the diagnosis at discharge seems to reflect a complication of, rather than the reason for, the procedure (e.g., different acute conditions or sepsis). Other limitations include a lack of data about important risk factors for mortality, like diabetes control, concomitant diseases. However, the errors inherent to a retrospective study may be balanced out by the large size of the population.

## 5. Conclusions

The number of lower-extremity amputations in diabetic patients in Poland increased substantially between the years 2010 and 2019, whereas the number of amputations in patients without diabetes was stable. This increase is due to the increasing number of patients with diabetes, seeing as the number of amputations/number of patients with diabetes ratio remains stable. The 30-day mortality rate after amputation was rather high and varied with different diagnoses at the discharge after procedures.

## Figures and Tables

**Table 1:** ICD-10 codes used for defining the diagnosis at discharge categories and ICD-9 codes used for defining amputation procedure.

Category	ICD-10 codes
Vascular	I70; R02
Bone/joint infection	M00; M01; M02; M03; M04; M05; M06; M07; M08; M09; M10; M11; M12; M13; M14; M15; M16; M17; M18; M19; M20; M21; M22; M23; M24; M25; M86; M87; M90
Soft tissue infection	A18.4; A20.1; A22.0; A26.0; A28.1; A30; A31.1; A32.0; A36.3; A43.1; A44.1; A46; A48.0; A51.3; L00; L01; L02; L03; L04; L05; L06; L07; L08; M60
Ulceration	L88; L89; L97; I83.0; I83.2
Trauma (including burns and frostbites)	S70; S71; S72; S73; S74; S75; S76; S77; S78; S79; S80; S81; S82; S83; S84; S85; S86; S87; S88; S89; S90; S91; S92; S93; S94; S95; S96; S97; 98; S99; T12; T13; T24; T25; T31; T33.6; T33.7; T33.8; T34.6; T34.7; T34.8; T35.5
Neoplasms	C00; C01; C02; C03; C04; C05; C06; C07; C08; C09; C10; C11; C12; C13; C14; C15; C16; C17; C18; C19; C20; C21; C22; C23; C24; C25; C26; C27; C28; C29; C30; C31; C32; C33; C34; C35; C36; C37; C38; C39; C40; C41; C42; C43; C44; C45; C46; C47; C48; C49; C50; C51; C52; C53; C54; C55; C56; C57; C58; C59; C60; C61; C62; C63; C64; C65; C66; C67; C68; C69; C70; C71; C72; C73; C74; C75; C76; C77; C78; C79; C80; C81; C82; C83; C84; C85; C86; C87; C88; C89; C90; C91; C92; C93; C94; C95; C96; C97; D00; D01; D02; D03; D04; D05; D06; D07; D08; D09; D10; D11; D12; D13; D14; D15; D16; D17; D18; D19; D20; D21; D22; D23; D24; D25; D26; D27; D28; D29; D30; D31; D32; D33; D34; D35; D36; D37; D38; D39; D40; D41; D42; D43; D44; D45; D46; D47; D48
Acute	I21; I22; I46; I74; J20; J21: J22; J46: J80; J95: J96; J98; N17; A48.3; R57; T81.1; T79.4; O08.3; O75.1; T78.0; T78.2; T80.5; T88.2; T88.6
Sepsis	A02.1; A20.7; A22.7; A24.1; A26.7; A32.7; A42.7; R09.0; R09.2; R57.8; A54.8; B00.7; B37.7; O75.3; T80.2; T81.4; T88.0; A40; A41; O85; B49
*Others*	
Diabetes	E10; E11; E12; E13; E14
Amputation	84.1; 84.10; 84.10; 84.101; 84.102; 84.103; 84.11; 84.11; 84.111; 84.113; 84.114; 84.119; 84.12; 84.12; 84.121; 84.122; 84.123; 84.124; 84.125; 84.129; 84.14; 84.15; 84.151; 84.31; 84.1; 84.10; 84.10; 84.102; 84.103; 84.11; 84.111; 84.113; 84.114; 84.119; 84.12; 84.121; 84.122; 84.123; 84.124; 84.125; 84.129; 84.14; 84.15; 84.151; 84.31; 84.1; 84.10; 84.102; 84.103; 84.11; 84.111; 84.113; 84.114; 84.119; 84.12; 84.121; 84.122; 84.123; 84.124; 84.125; 84.129; 84.14; 84.15; 84.151; 84.31; 84.1; 84.10; 84.101; 84.102; 84.103; 84.11; 84.111; 84.113; 84.114; 84.119; 84.12; 84.121; 84.122; 84.123; 84.124; 84.125; 84.129; 84.14; 84.15; 84.151; 84.31; 84.1; 84.10; 84.10; 84.102; 84.103; 84.11; 84.111; 84.113; 84.114; 84.119; 84.12; 84.121; 84.122; 84.123; 84.124; 84.125; 84.129; 84.14; 84.15; 84.151; 84.31; 84.1; 84.101; 84.102; 84.103; 84.11; 84.111; 84.113'; 4.114; 84.119; 84.12; 84.121; 84.122; 84.123; 84.124; 84.125; 84.129; 84.14; 84.15; 84.151; 4.31; 84.1; 84.101; 84.10; 84.103; 84.11; 84.111; 84.113; 84.114; 84.119; 84.121; 84.122; 84.123; 84.124; 84.125; 84.129; 84.14; 84.15; 84.151; 84.31; 84.1; 84.10; 84.10; 84.102; 84.103; 84.11; 84.111; 84.113; 84.114; 84.119; 84.12; 84.121; 84.122; 84.123; 84.124; 84.125; 84.129; 84.14; 84.151; 84.31; 84.1; 84.10; 84.101; 84.102; 84.103; 84.11; 84.111; 84.113; 84.114; 84.119; 84.12; 84.121; 84.122; 84.123; 84.124; 84.125; 84.129; 84.14; 84.15; 84.151; 84.31; 84.1; 84.10; 84.101; 84.102; 84.103; 84.11; 84.111; 84.113; 84.114; 84.119; 84.12; 84.121; 84.122; 84.123; 84.124; 84.125; 84.129; 84.14; 84.15; 84.151; 84.31

**Table 2:** Thirty-day mortality according to diagnosis at discharge categories in patients with and without diabetes.

Category	Patients with diabetes		Patients without diabetes	
	Number of amputations	Number of deaths (%)	Number of amputations	Number of deaths (%)
Vascular	16515	1383 (8.37)	14785	1421 (9.61)
Bone/joint infection	3030	105 (3.46)	2533	66 (2.6)
Soft tissue infection	7918	47 (5.27)	3871	275 (7.1)
Ulceration	1670	111 (6.64)	868	79 (9.1)
Trauma (including burns and frostbites)	334	20 (5.99)	3470	91 (2.62)
Neoplasms	262	11 (4.2)	847	19 (2.24)
Acute	450	99 (22.0)	700	147 (21.0)
Sepsis	100	34 (34.0)	69	20 (28.99)
Others	948	104 (10.97)	4393	149 (3.39)
Diabetes	34433	1635 (4.74)	—	—

## Data Availability

All data from the database can be obtained upon reasonable request from Marta Raczynska (m.raczynska@mz.gov.pl).

## References

[1] Lin X., Xu Y., Pan X. (2020). Global, regional, and national burden and trend of diabetes in 195 countries and territories: an analysis from 1990 to 2025. *Scientific Reports*.

[2] Towpik I., Walicka M., Marcinkowska K. (2020). Epidemiology of diabetes in Poland in 2014-2017. *Clinical Diabetology*.

[3] Sutkowska E. (2012). Frequency of foot pathologies among patients with diabetes in Wroclaw. *Clinical Diabetology*.

[4] Zhang Y., Lazzarini P. A., McPhail S. M., van Netten J. J., Armstrong D. G., Pacella R. E. (2020). Global disability burdens of diabetes-related lower-extremity complications in 1990 and 2016. *Diabetes Care*.

[5] Lazzarini P. A., Clark D., Derhy P. H. (2011). What are the major causes of lower limb amputations in a major Australian teaching hospital? The Queensland Diabetic Foot Innovation Project, 2006 – 2007. *Journal of foot and ankle research*.

[6] Dunbar G. L., Hellenberg D. A., Levitt N. S. (2015). Diabetes mellitus and non-traumatic lower extremity amputations in four public sector hospitals in Cape Town, South Africa, during 2009 and 2010. SAMJ, S. *African Medical Journal*.

[7] Sinha R., van den Heuvel W. J., Arokiasamy P. (2011). Factors affecting quality of life in lower limb amputees. *Prosthetics and Orthotics International*.

[8] Sahu A., Sagar R., Sarkar S., Sagar S. (2016). Psychological effects of amputation: a review of studies from India. *Industrial Psychiatry Journal*.

[9] Gurney J. K., Stanley J., Rumball-Smith J., York S., Sarfati D. (2018). Postoperative death after lower-limb amputation in a national prevalent cohort of patients with diabetes. *Diabetes Care*.

[10] Cascini S., Agabiti N., Davoli M. (2020). Survival and factors predicting mortality after major and minor lower-extremity amputations among patients with diabetes: a population-based study using health information systems. *BMJ Open Diabetes Research & Care*.

[11] (2013). *Open Source Epidemiologic Statistics for Public Health*.

[12] Lopez-de-Andres A., Jiménez-García R., Aragón-Sánchez J. (2015). National trends in incidence and outcomes in lower extremity amputations in people with and without diabetes in Spain, 2001-2012. *Diabetes Research and Clinical Practice*.

[13] Lopez-Bastida J., Boronat M., Moreno J. O., Schurer W. (2013). Costs, outcomes and challenges for diabetes care in Spain. *Globalization and Health*.

[14] Claessen H., Avalosse H., Guillaume J. (2018). Decreasing rates of major lower-extremity amputation in people with diabetes but not in those without: a nationwide study in Belgium. *Diabetologia*.

[15] Buckley C. M., O’Farrell A., Canavan R. J. (2012). Trends in the incidence of lower extremity amputations in people with and without diabetes over a five-year period in the Republic of Ireland. *PLoS One*.

[16] Aziz F., Reichardt B., Sourij C. (2020). Epidemiology of major lower extremity amputations in individuals with diabetes in Austria, 2014-2017: a retrospective analysis of health insurance database. *Diabetes Research and Clinical Practice*.

[17] Hughes W., Goodall R., Salciccioli J. D., Marshall D. C., Davies A. H., Shalhoub J. (2020). Editor's Choice - Trends in lower extremity amputation incidence in European Union 15+ countries 1990-2017. *European Journal of Vascular and Endovascular Surgery*.

[18] Wierzba W., Krasnodębski P., Śliwczyński A., Karnafel W. (2020). Geographic variability of major non-traumatic lower limb amputations in diabetic and non-diabetic patients in Poland. *Annals of Agricultural and Environmental Medicine*.

[19] Monteiro-Soares M., Vale-Lima J., Martiniano J., Pinheiro-Torres S., Dias V., Boyko E. J. (2021). A systematic review with meta-analysis of the impact of access and quality of diabetic foot care delivery in preventing lower extremity amputation. *Journal of Diabetes and its Complications*.

[20] Ramos Vieira Santos I. C., Carvalho E. F., Souza W. V., Albuquerque E. C. (2015). Factors associated with diabetic foot amputations. *Jornal Vascular Brasileiro*.

[21] Lin C., Liu J., Sun H. (2020). Risk factors for lower extremity amputation in patients with diabetic foot ulcers: a meta-analysis. *PLoS One*.

[22] Dunbar G. L., Hellenberg D. A., Levitt N. S. (2015). Diabetes mellitus and non-traumatic lower extremity amputations in four public sector hospitals in Cape Town, South Africa, during 2009 and 2010. *South African Medical Journal*.

[23] Viswanathan V., Kumpatla S. (2011). Pattern and causes of amputation in diabetic patients--a multicentric study from India. *The Journal of the Association of Physicians of India*.

[24] Fosse S., Hartemann-Heurtier A., Jacqueminet S., Ha Van G., Grimaldi A., Fagot-Campagna A. (2009). Incidence and characteristics of lower limb amputations in people with diabetes. *Diabetic Medicine*.

[25] Gurney J. K., Stanley J., York S., Rosenbaum D., Sarfati D. (2018). Risk of lower limb amputation in a national prevalent cohort of patients with diabetes. *Diabetologia*.

[26] Lin C. S., Chang C. C., Lee Y. W. (2019). Adverse outcomes after major surgeries in patients with diabetes: a multicenter matched study. *Journal of Clinical Medicine*.

[27] Ata A., Valerian B. T., Lee E. C., Bestle S. L., Elmendorf S. L., Stain S. C. (2010). The effect of diabetes mellitus on surgical site infections after colorectal and noncolorectal general surgical operations. *The American Surgeon*.

[28] Wallaert J. B., Nolan B. W., Adams J. (2012). The impact of diabetes on postoperative outcomes following lower-extremity bypass surgery. *Journal of Vascular Surgery*.

[29] Hoffstad O., Mitra N., Walsh J., Margolis D. J. (2015). Diabetes, lower-extremity amputation, and death. *Diabetes Care*.

[30] Schofield C. J. (2006). Mortality and hospitalization in patients after amputation: a comparison between patients with and without diabetes. *Diabetes Care*.

[31] Kennedy G., McGarry K., Bradley G., Harkin D. W. (2019). All-cause mortality amongst patients undergoing above and below knee amputation in a regional vascular centre within 2014-2015. *The Ulster Medical Journal*.

